# Patient-specific approach using data fusion and adversarial training for epileptic seizure prediction

**DOI:** 10.3389/fncom.2023.1172987

**Published:** 2023-05-04

**Authors:** Yong Yang, Xiaolin Qin, Han Wen, Feng Li, Xiaoguang Lin

**Affiliations:** ^1^Chengdu Institute of Computer Application, Chinese Academy of Sciences, Chengdu, Sichuan, China; ^2^Chongqing Institute of Green and Intelligent Technology, Chinese Academy of Sciences, Chongqing, China; ^3^Chongqing School, University of Chinese Academy of Sciences, Chongqing, China; ^4^Department of Neurology, The First Affiliated Hospital of Chongqing Medical University, Chongqing, China

**Keywords:** seizure prediction, EEG, ECG, data fusion, adversarial training

## Abstract

Epilepsy is the second common neurological disorder after headache, accurate and reliable prediction of seizures is of great clinical value. Most epileptic seizure prediction methods consider only the EEG signal or extract and classify the features of EEG and ECG signals separately, the improvement of prediction performance from multimodal data is not fully considered. In addition, epilepsy data are time-varying, with differences between each episode in a patient, making it difficult for traditional curve-fitting models to achieve high accuracy and reliability. In order to improve the accuracy and reliability of the prediction system, we propose a novel personalized approach based on data fusion and domain adversarial training to predict epileptic seizures using leave-one-out cross-validation, which achieves an average accuracy, sensitivity and specificity of 99.70, 99.76, and 99.61%, respectively, with an average error alarm rate (FAR) of 0.001. Finally, the advantage of this approach is demonstrated by comparison with recent relevant literature. This method will be incorporated into clinical practice to provide personalized reference information for epileptic seizure prediction.

## 1. Introduction

Epilepsy is a chronic disorder in which abnormal neuronal discharges occur suddenly, causing temporary brain dysfunction. According to the World Health Organization (WHO), there are approximately 70 million people with epilepsy worldwide, and epilepsy has become the second common neurological disorder after headache (Rasheed et al., 2021). In addition, before an epileptic seizure occurs, there are abnormalities in the EEG (electroencephalogram) and ECG (electrocardiogram) that make it possible to predict epileptic seizures (Rakhade and Jensen, 2009). If the seizure can be predicted in advance, there is enough time for the medical staff to intervene with the patient’s medication or to apply electrical stimulation treatment to protect the patient from side effects.

Electroencephalogram is a method of recording brain activity using electrophysiological indicators (Zhang et al., 2020) and is the overall reflection of the electrophysiological activity of brain nerve cells in the cerebral cortex. In recent years, the use of EEG for seizure detection and prediction has received widespread attention from the academic community. Based on the dynamic characteristics of the EEG signal, brain activity in epileptic situations can be classified into four phases (Jana and Mukherjee, 2021a,b): preictal, ictal, postictal, and interictal. Studies have shown that the transition from interictal to ictal state in patients with epilepsy is not instantaneous, but rather goes through a transitional period, so accurate and efficient identification of the preictal state becomes the key to epileptic seizure prediction (Yuan et al., 2018). According to previous research, ECG signal analysis can help predict epileptic seizures. Some studies found significant changes along with the preictal parts of ECG signals by analyzing the parameters of heart rate variability and comparing them statistically in the preictal and interictal parts. These changes indicate high sympathetic activity and occur especially 5 min before the onset of seizures. Therefore, ECG signal analysis can be a beneficial tool for predicting epileptic seizures (Seifi et al., 2022).

The pathogenesis of epilepsy is complex and the types of epileptic seizures are diverse. In addition, since the patient’s status during each seizure cycle is different, such as physical condition, pathogenesis, seizure intensity, seizure type, environmental influences, mood, etc., these multiple factors lead to the preictal and interictal periods before each ictal having different characteristics. Therefore, in the same patient, the signal characteristics of the preictal period and the interictal period preceding each ictal may be the same or different (Babb et al., 1987; Fisher et al., 2017).

Most of the existing epileptic seizure prediction methods focus on the patient-specific scenario (Detti et al., 2018) which refers to predicting a patient’s epileptic seizure by learning from his own historical records, this method is easy to implement and has high prediction accuracy, and is favored by a wide range of researchers, but the accuracy is unstable, that is, large differences in results between patients, so the traditional methods need further improvement.

To eliminate the negative effect of data distribution shift, DANN (domain-adversarial training of neural networks) (Ganin et al., 2016; Yu et al., 2019; Matsuura and Harada, 2020) used marginal distribution alignment to eliminate data differences between subjects, and MADA (multi-adversarial domain adaptation) (Pei et al., 2018) used conditional distribution alignment to achieve better invariant feature learning. However, these proposed methods were only for eliminating differences between patients and did not address the negative effect of multiple seizures in a patient to predict epilepsy, resulting in unstable prediction results on the patient-specific scenario.

To further improve the prediction accuracy and reliability, data fusion is an effective way; in actual EEG acquisition, simultaneous acquisition of ECG data can meet the requirement of data fusion; in addition, there is increasing evidence that the nervous system plays an important role in regulating cardiac function, and seizure-onset arrhythmias have been shown to be the result of autonomic imbalance induced by seizure activity. Studies have shown that seizures can be predicted using the ECG (Moridani and Farhadi, 2017; Ufongene et al., 2020; Costagliola et al., 2021), so combining the EEG with the ECG signal not only improves the accuracy of seizure prediction, but also allows better detection and understanding of brain-heart interactions for monitoring and treatment of potential arrhythmias.

Deep learning (DL), a branch of machine learning (ML), has become increasingly popular in recent years. DL has been shown to outperform traditional ML in many areas, and through the efforts of researchers, the DL method has been extensively applied to the seizure prediction task and has achieved promising results (Jana and Mukherjee, 2021a,b; Usman et al., 2021; Zhang et al., 2021). However, most of the current methods use only EEG signals for seizure prediction and do not consider the effect of multimodal data on prediction performance.

In studies using simultaneous EEG and ECG signals for epileptic seizure prediction, Phomsiricharoenphant et al. (2014) used the Hilbert Huang Transform (HHT) to extract the instantaneous mean frequency from EEG and the R-R interval from ECG simultaneously for epileptic seizure prediction independently, the possibility of using EEG and ECG for seizure prediction was first explored; Hoyos-Osorio et al. (2016) used EEG features extracted by discrete wavelet transform and ECG features extracted by calculating heart rate changes to predict epileptic seizures independently; Billeci et al. (2019) used a combination of EEG and ECG to extract synchronous mode and time-frequency features from EEG, and extracted inter-beat (RR) information from ECG using recurrence quantification analysis to predict epileptic seizures independently, and used SVM classifier to classify preictal and interictal phases by combining features extracted from both signals. Most of these methods analyze the EEG and ECG data independently and derive the final prediction results independently, without sufficiently considering the combined EEG and ECG data for seizure prediction, and the prediction performance could be further improved.

Therefore, in order to improve prediction accuracy and reliability, eliminate the negative impact of data differences of each seizure in a patient, it is necessary to develop a personalized epileptic prediction approach with data fusion and adversarial training method for specific patient.

The rest of this paper is organized as follows. In section “2. Scalp EEG dataset and methods,” the Scalp EEG dataset and the epileptic seizure prediction method based on data fusion and adversarial training is described in details. In section “3. Results and discussion,” results and discussion on benchmark dataset are provided. In the end, some conclusions are given in section “4. Conclusion.”

## 2. Scalp EEG dataset and methods

### 2.1. Scalp EEG data

The proposed approach is evaluated on a benchmark dataset published by the University of Siena, Italy. This dataset consists of 9 males (36–71), 5 females (20–58), collected from scalp electrodes using the international 10–20 system at 512 Hz sampling rate, most of the data include EEG and ECG signals simultaneously. Personal information and epileptic seizure information are shown in Table 1.

**TABLE 1:** Siena scalp EEG dataset information.

Patient_id	Age_years	Gender	Seizure	EEG channel	Number seizures	Time(s)
PN00	55	Male	IAS	29	5	198
PN01	46	Male	IAS	29	2	809
PN03	54	Male	IAS	29	2	752
PN05	51	Female	IAS	29	3	359
PN06	36	Male	IAS	29	5	722
PN07	20	Female	IAS	29	1	523
PN09	27	Female	IAS	29	3	410
PN10	25	Male	FBTC	20	10	1,002
PN11	58	Female	IAS	29	1	145
PN12	71	Male	IAS	29	4	246
PN13	34	Female	IAS	29	3	519
PN14	49	Male	WIAS	29	4	1,408
PN16	41	Female	IAS	29	2	303
PN17	42	Male	IAS	29	2	308

For each patient, the minimum length of the preictal and interictal periods is set at 5 min, the preictal and interictal periods before each seizure as a unit and recorded as an episode; we evaluated patients who had at least three seizures in the EEG recordings. This is because less than three preictal and interictal periods cause an overfitting problem in training. Taking all these restrictions into account, eight patients are available, such as P00, P05, P06, P09, P10, P12, P13, and P14.

### 2.2. Epileptic seizure prediction method based on data fusion and adversarial training

**FIGURE 1:**
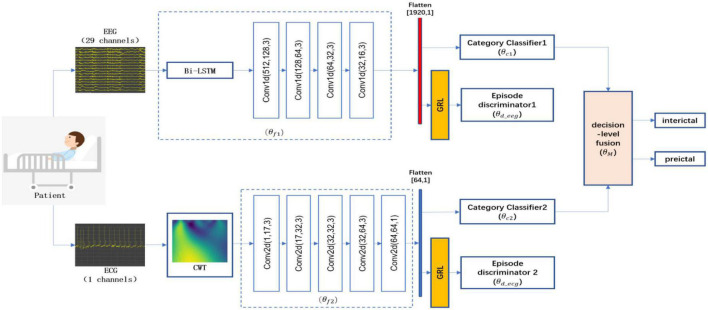
Flowchart of the epileptic seizure prediction model. A block diagram showing the components of the prediction model, in which the electroencephalogram (EEG) and electrocardiogram (ECG) signals are feature extracted and classified, respectively, and then the classification results are post-processed by a decision-level fusion strategy to obtain the final prediction results.

The proposed epileptic seizure prediction method explores the data fusion strategy of EEG and ECG signal and eliminates the negative impact of data difference of each seizure in a patient. It consists of three sections (Figure 1): (1) EEG feature extraction and classification module; (2) ECG feature extraction and classification module; and (3) decision level fusion module. The EEG feature extraction and classification module is composed of a long short-term memory network (LSTM) and four layers of 1-D CNN, and the ECG feature extraction and classification module is composed of five layers of 2-D CNN, the decision-level fusion module combines the output results of the EEG and ECG classification modules according to dynamic weight, so that the EEG classification module and the ECG classification module achieve prediction performance complementarity. In addition, to eliminate the time-varying of each episode in some epilepsy patients, the adversarial training is used, which increases the ability of extracting the invariant features.

#### 2.2.1. Data pre-processing

Data pre-processing is an important step before the feature extraction stage, and perfect data pre-processing will guarantee the high accuracy in the later stage; in pre-processing, firstly, useless electrodes must be removed, followed by filtering, because the frequency range from 0.1 to 70 Hz contains most of the epilepsy-related information (Das et al., 2020), we set the bandpass filter range as follows: low-frequency filtering is set to 0.1 Hz, high frequency filtering is set to 70 Hz, and a notch filter is set from 48 to 52 Hz to eliminate power frequency interference; in addition, to improve the accuracy of the algorithm, we use min-max regularization technology (Sola and Sevilla, 1997) to regularize the data, the data is between 0 and 1 after regularization. The min-max regularization method is shown below:


(1) $$X_{\text{normal}}=\frac{X_{\text{i}}-X_{\min}}{X_{\max}-X_{\min}}$$

Where,*X*_i_ is the original data, *X*_normal_ is the data after regularization, *X*_min_ and *X*_max_ is the minimum and maximum values of the original data. After regularizing the EEG data, all channel data are selected (30 channels are included in the Siena scalp EEG dataset after removal of useless electrodes, of which 29 EEG channels, 1 ECG channel), to facilitate later feature extraction and classification, the EEG and ECG data are segmented separately; the data are segmented using moving window analysis, with a window length of 1 s and 0% overlapping; For labeling the data segment, when the data segments are in the interictal state, the segment data is labeled as 0, and when the data segments are in the preictal state, the segmented data is labeled as 1; For labeling each episode in a patient, the episodes are labeled from 0 to N-1 (N is the number of episodes in a patient). Finally, the EEG dataset and the ECG dataset are formed.

In order to extract the spectral characteristics of ECG data, we adopt a continuous wavelet transform, the wavelet scale is 32 and the mother wavelet is “mexh,” so as to obtain the effective characterization signal of the original signal in the frequency domain (Shukla et al., 2022), and the continuous wavelet transform is shown below:


(2) $$W\,T\,f\,\left(a,b\right)=\frac{1}{\sqrt{a}}\,\int_{-\infty }^{+\infty }f\,\left(t\right)\,\Psi^{*}\left(\frac{t-b}{a}\right)\,d\,t$$

Where, WT represents the wavelet transform, _*f(t)*_ is the original signal; _*a*_ (_*a*_>0), _*b*_ are scale parameters and translation parameters, respectively; _Ψ((t–b)/a)_ is sub-wave, it is the scaling and translation of the mother wavelet.

After pre-processing, the pre-processed data is fed into the feature extraction and classification module for feature extraction and classification.

#### 2.2.2. Feature extraction

##### 2.2.2.1. Bi-LSTM+1D CNN network

We use the Bi-LSTM network (Gers et al., 1999; Tsiouris et al., 2018; Cheng et al., 2021) to extract features from the EEG data. Bi-LSTM network is a kind of RNN, where each block is composed of two LSTM blocks, respectively, processing forward and backward EEG signal, the forward module processes the time feature vector along the positive sequence of the time series, while the backward transmission block processes the same time feature in the reverse sequence of the time series. The output of each block is a combination of processing results in two opposite directions, the Bi-LSTM network not only processes the current feature extraction, but also extracts future features, so it can effectively improve the recognition accuracy. Bi-LSTM+1D CNN network is mainly to extract features of EEG data, then the extracted features are classified by the two full connection layer networks.

##### 2.2.2.2. 2D CNN network

Since the ECG signal does not change significantly in the time domain, there are subtle feature changes in the frequency domain, therefore the feature extraction and classification of the ECG data adopts 2D image format, due to the convolution, pooling and other operations contained in the CNN network (Khan et al., 2018; Liu et al., 2020), it is very applicable to process 2D image, so the feature extraction of the ECG signal adopts the 2D CNN network. Finally, the two full connection layers are used to classify the extracted ECG features.

### 2.3. Data fusion strategy

Data fusion is the combination of data and information from multi-sensor information sources, the purpose is to timely mine hidden information from big data, so how to reasonably and effectively integrate and intelligently process big data is a problem of data fusion. According to different stages of data fusion, data fusion is divided into three categories: data-level fusion, feature-level fusion and decision-level fusion (Balasubramaniam and Ananthi, 2014; Javed et al., 2014; Versaci et al., 2022).

For data-level fusion, the pre-processed raw data from several different sources are combined according to certain rules, e.g., the EEG signal and the ECG signal are combined; note that the combined data must keep the sampling rate consistent and the data time aligned.

For feature level fusion, to alleviate the problem of inconsistencies between the original data in each mode, the representation of the features can be taken separately from each mode, and then the extracted feature is combined to form a new feature, which is provided for later data classification.

In decision level fusion, the DL model trains the data from different modes and then fuses the output results for later data classification; decision level fusion mainly uses rules such as the mean method, voting method and ensemble learning method.

In our work, to further improve the performance of the DL model, we proposed a novel network consisting of two branches (EEG feature extraction and classification branch, ECG feature extraction and classification branch), each branch is an independent network, and the output result of the network is the weighted average of each branch (Saranya et al., 2021):


(3) $$y=\sum_{i=1}^{L}w_{i}d_{i}$$

Where, _*w_i_*_ is the weight, _*w*_1_ + *w*_2_ + *w*_L_=1,_ *L* is the number of branch network (*L* = 2), _*d_i_*_ is the prediction results of the *i*th branch network. For weighted averages _*w_i_*_, can be fixed to 0.5, however, after such a fixed setting, the classification error rate of the branch network cannot affect the weights of the branch, resulting in no improvement in the classification accuracy, so we use the classification error rate of the branch to dynamically adjust the weight of the branch, the weights are calculated as follows:


(4) $$w_{1}=1+a\,r\,c\,t\,a\,n\,\left(\frac{1-e\,r\,r\,1}{e\,r\,r\,1}\right)$$

(5) $$w_{2}=1+a\,r\,c\,t\,a\,n\,\left(\frac{1-e\,r\,r\,2}{e\,r\,r\,2}\right)$$

(6) $$W_{1}=\frac{w_{1}}{w_{1}+w_{2}}$$

(7) $$W_{2}=\frac{w_{2}}{w_{1}+w_{2}}$$

Where, _*err1*_ and _*err2*_ are the classification error rate of the two branch networks, _*w_1_*_ and_*w_2_*_ are the weights of each branch network, _*W_1_*_ and _*W_2_*_ are the normalized value of _*w_1_*_ and_*w_2_*,__*W_1_ + W_2_=1*_. By dynamically weighting the output of each branch, a network model with better performance, stronger generalization ability, and higher stability is constructed.

### 2.4. Adversarial training

For a particular patient, the EEG and ECG data are time-varying, resulting in each episode being different, in order to eliminate the negative impact of data distribution shift between episodes, we adopt adversarial training (Ganin et al., 2016), which aims to improve the generalization ability and prediction accuracy of the model. For the EEG data, the features are extracted by feature extraction networks _*G*_*f*1__, then, the features are fed into the episode discriminator 1; For the ECG data, the features are extracted by feature extraction networks _*G_*f*2_*_, then, the features are fed into the episode discriminator 2; the losses of episode discriminator 1 and 2 are as follows:


(8) $$L_{d\,\_\,e\,e\,g}=\frac{1}{N}\,\sum_{x_{e\,e\,g\,\_\,i}\in D_{e\,e\,g}}L\,\left(G_{d\,\_\,e\,e\,g}\,\left(G_{f\,1}\,\left(x_{e\,e\,g\,\_\,i}\right)\right),d_{i}\right)$$

(9) $$L_{d\,\_\,e\,c\,g}=\frac{1}{N}\,\sum_{x_{e\,c\,g\,\_\,i}\in D_{e\,c\,\text{g}}}L\,\left(G_{d\,\_\,e\,c\,g}\,\left(G_{f\,2}\,\left(x_{e\,c\,g\,\_\,i}\right)\right),d_{i}\right)$$

Where, _*L*_ is the cross entropy loss function, *G*_*f*1_ is the EEG feature extraction network, *G*_*f*2_ is the ECG feature extraction network, *G*_*d*_*eeg*_ is the EEG episode discriminator, *G*_*d*_*ecg*_ is the ECG episode discriminator, *d*_*i*_ is the episode label, *x*_*eeg*_*i*_ and *x*_*ecg*_*i*_ are the EEG and ECG data sample, *D*_*eeg*_ and *D*_*ec*g_ are the dataset of EEG data and ECG data in a patient, _*N*_ is the number of samples.

### 2.5. Training details

The proposed approach adopts data fusion strategy, the loss function of classifier is:


(10) $$L_{c}=\frac{1}{N}\sum_{x_{e\,e\,g-i}\in D_{e\,e\,g;e\,c\,g-i}\in D_{e\,c\,g}}L(M(G_{c\,1}\left(G_{f\,1}\left(x_{e\,e\,g-i}\right)\right),$$

$$G_{c\,2}\left(G_{f\,2}\left(x_{e\,c\,g-i}\right)\right)),y_{i})$$

Where, _*L*_ is the cross entropy loss function, *G*_*c*1_ is the EEG category classifier, *G*_*c*2_ is the ECG category classifier, *y*_*i*_ is the category label, and _*M*_is data fusion strategy:


(11) $$M=W_{1}*G_{c\,1}(G_{f\,1}\left(x_{e\,e\,g\,\_\,i}\right)+W_{2}*G_{c\,2}(G_{f\,2}\left(x_{e\,c\,g\,\_\,i}\right)$$

Where, _*W_1_*_ and _*W_2_*_ are weights of each branch which can be learnt by the training.

We propose an adversarial training strategy to jointly train all the loss functions:


(12) $$L_{s\,u\,m}=L_{c}-\lambda \times \left(L_{d\,\_\,e\,e\,g}+L_{d\,\_\,e\,c\,g}\right)$$

Where, _λ_ = 0.1; During optimization, episode discriminator 1 and episode discriminator 2 are trained by a special layer called Gradient Reversal Layer (GRL), which connects feature extraction network and episode discriminator, this GRL is omitted during forward propagation, the gradient is reversed in backpropagation. Finally, we search the optimal parameters $\overset{\wedge }{{\text{θ}}_{f1}}\overset{\wedge }{{\text{θ}}_{f2}}\overset{\wedge }{{\text{θ}}_{c1}}\overset{\wedge }{{\text{θ}}_{c2}}\overset{\wedge }{\underset{\mathrm{d\_egg}}{\text{θ}}}\;\overset{\wedge }{\underset{\mathrm{d\_ecg}}{\text{θ}}}\;\overset{\wedge }{\underset{M}{\text{θ}}}$ to meet the following requirements:


(13) $$\left(\overset{\wedge }{\underset{f\,1}{\theta }},\overset{\wedge }{\underset{f\,2}{\theta }},\overset{\wedge }{\underset{c\,1}{\theta }},\overset{\wedge }{\underset{c\,2}{\theta }},\overset{\wedge }{\underset{M}{\theta }}\right)=\arg\min_{\theta_{f\,1},\theta_{f\,2},\theta_{c\,1},\theta_{c\,2},\theta_{M}}L_{\text{sum}}(\theta_{f\,1},\theta_{f\,2},\theta_{c\,1},\theta_{c\,2},\theta_{d\,\_\,e\,e\,g},\theta_{d\,\_\,e\,c\,g})$$

(14) $$\left(\overset{\wedge }{\underset{d\,\_\,e\,e\,g}{\theta }},\overset{\wedge }{\underset{d\,\_\,e\,c\,g}{\theta }}\right)=\arg\max_{\theta_{d\,\_\,e\,e\,g},\theta_{d\,\_\,e\,c\,g}}L_{\text{sum}}\,\left(\theta_{f\,1},\theta_{f\,2},\theta_{c\,1},\theta_{c\,2},\theta_{d\,\_\,e\,e\,g},\theta_{d\,\_\,e\,c\,g}\right)$$

### 2.6. Evaluation

Leave-one-out cross-validation is used in the training and test phases, where the number of training sessions for a given patient is equal to the number of episodes. During each training session, except for one episode which is used in the test, all other episodes participated in the training process, and the process is repeated by changing the patient’s episode.

#### 2.6.1. Experimental environment

The experimental environment is: Windows 10 operation system, the program language is Python 3.7.4, and the DL framework is Pytorch (its version is 11.1). The graphics card is: GeForce RTX 3060.

#### 2.6.2. Experimental parameters

This experiment uses two independent training branches, including LSTM+CNN network for EEG data and 2-D CNN network for ECG data, training epoch is set to 100 times and batch size is set to 128. Cross entropy loss function and Center loss function were combined as the loss function, the model uses the Adam optimizer and learning rate is set to 0.001, center loss function is optimized using the SGD optimizer, and learning rate is set to 0.05. For the setting of hyperparameters in the experiment, we use grid search method.

#### 2.6.3. Evaluate metrics

The experiment used accuracy (ACC), sensitivity (SN), and specificity (SP) to quantify the performance of the algorithm (Prasanna et al., 2021).


(15) $$A\,C\,C=\frac{T\,P+T\,N}{T\,P+T\,N+F\,P+F\,N}$$

(16) $$S\,N=\frac{T\,P}{T\,P+F\,N}$$

(17) $$S\,P=\frac{T\,N}{T\,P+T\,N}$$

Where, TP (True Positive): the sample that is positive is judged to be positive, TN (True Negative): the sample that is negative is judged to be negative, FP (False Positive): the negative sample is considered positive, FN (False Negative): the sample that is positive is judged to be negative.

To evaluate the prediction accuracy of the forecasting system, the false alarm rate is measured as follows (Cheng et al., 2021):


(18) $$F\,A\,R=\frac{N_{a\,l\,a\,r\,m}}{N_{w\,o}}$$

Where, _*N_alarm_*_ represents the number of time windows for alarms in the interictal, _*N_wo_*_ represents the number of time windows in interictal.

## 3. Results and discussion

### 3.1. Results of the proposed approach

To evaluate the advantages and disadvantages of the proposed approach, we selected eight patients with a number of epileptic seizures greater than or equal to three from the Siena scalp EEG dataset, the results of each epilepsy patient are shown in Table 2.

**TABLE 2:** Test results on Siena scalp dataset.

Patient number	ACC (%)	SN (%)	SP (%)	FAR
P00	99.39	99.62	98.89	0.004
P05	100	100	100	0
P06	99.63	99.34	99.82	0.002
P09	99.12	99.45	98.97	0.002
P10	100	100	100	0
P12	100	100	100	0
P13	100	100	100	0
P14	99.42	99.69	99.21	0.002
Average	99.70 ( ± 0.35)	99.76 ( ± 0.27)	99.61 ( ± 0.5)	0.001 ( ± 0.037)

Since there is very little literature on seizure prediction using the Siena scalp EEG dataset, we set the preictal period length to *T* = 300 s (5 min), the same preictal period length setting proposed in the literature (Detti et al., 2020), and compared with the literature (Phomsiricharoenphant et al., 2014; Hoyos-Osorio et al., 2016; Billeci et al., 2019; Detti et al., 2020). The comparison results of the related methods are shown in Table 3. The results show that the proposed approach has an advantage in all test metrics.

**TABLE 3:** Comparison results of related methods.

Methods	ACC (%)	SN (%)	SP (%)	FAR (%)
Phomsiricharoenphant et al., 2014	88.32 ( ± 2.28)	86.52 ( ± 2.40)	90.72 ( ± 2.43)	0.085 ( ± 0.93)
Hoyos-Osorio et al., 2016	90.89 ( ± 2.19)	91.36 ( ± 1.69)	89.70 ( ± 2.58)	0.068 ( ± 0.91)
Billeci et al., 2019	91.32 ( ± 2.56)	94.26 ( ± 1.14)	86.58 ( ± 3.73)	0.057 ( ± 0.44)
Detti et al., 2020	100 ( ± 0)	98.88 ( ± 2.01)	–	–
Ours	99.70 ( ± 0.35)	99.76 ( ± 0.27)	99.61 ( ± 0.5)	0.001 ( ± 0.037)

In order to compare performance with other approaches, we used *t*-test in the case of normal distributions to calculate the *p*-values of the accuracy between literatures (Phomsiricharoenphant et al., 2014; Hoyos-Osorio et al., 2016; Billeci et al., 2019; Detti et al., 2020) and the proposed approach, the results are 1.34E-09, 2.22E-08, 5.94E-08, 0.029, respectively, all less than 0.05, therefore, the approach in this paper significantly outperforms the contrastive literatures.

### 3.2. Comparison of the methods of data fusion

**FIGURE 2:**
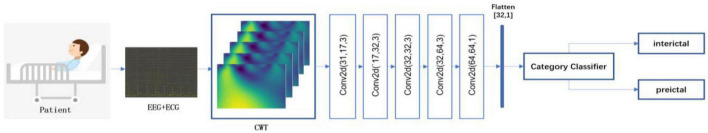
Flowchart of the data-level fusion epileptic seizure prediction model. This method first combines EEG and ECG signals, and then performs feature extraction and classification.

**FIGURE 3:**
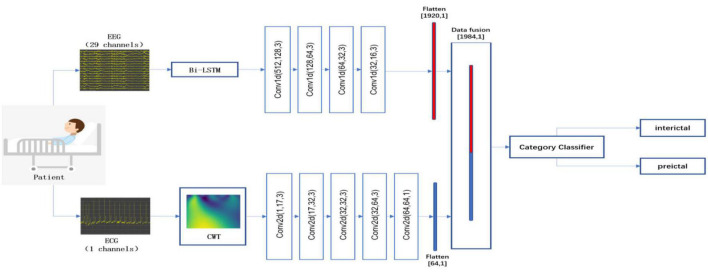
Flowchart of the feature-level fusion epileptic seizure prediction model. This method performs pre-processing and feature extraction from EEG and ECG signals independently, and then fuses and classifies the features of the EEG and ECG signals.

To further demonstrate the superiority of the decision-level fusion method proposed in this paper, we compare the performance of the data-level fusion and feature-level fusion approaches. The two approaches are shown in Figures 2, 3.

The comparison results of the three data fusion approaches are shown in Table 4.

**TABLE 4:** Comparison results of three data fusion methods.

Data fusion method	ACC (%)	SN (%)	SP (%)	FAR
Data-level fusion	95.33 ( ± 1.17)	96.12 ( ± 1.50)	92.19 ( ± 1.30)	0.018 ( ± 0.69)
Feature-level fusion	92.31 ( ± 1.50)	90.35 ( ± 2.22)	94.16 ( ± 0.70)	0.032 ( ± 0.80)
Decision-level fusion	99.70 ( ± 0.35)	99.76 ( ± 0.27)	99.61 ( ± 0.5)	0.001 ( ± 0.037)

In order to compare performance with other data fusion approaches, we used *t*-test in the case of normal distributions to calculate the *p*-values of the accuracy between data-level/feature-level fusion approach and the proposed approach, the results are 8.29E-08, 1.92E-09, respectively, all less than 0.05, therefore, the approach in this paper significantly outperforms the contrastive data fusion approaches.

Through comparison, it can be seen that the effect of the decision-level fusion approach is optimal, followed by the data-level fusion approach, the feature-level fusion approach is the worst, analyze its reasons, there are three reasons, first, EEG and ECG signals can be used as complementary to each other in epileptic seizure prediction, through independent feature extraction of EEG and ECG signals, the shortcomings of the extraction features of a single network can be effectively avoided; second, for the feature-level fusion approach, the two independent branch networks are used for EEG and ECG signal feature extraction, but the separately extracted feature dimensions are limited by artificial settings, the specific setting is still unknown how much is the best state, so the result is the worst; third, for the data level fusion approach, the EEG and ECG data are combined in the same mode, then the feature extraction is performed by a unified network, which ignores the diversity of the data, resulting in the information from the multimodal data is not extracted and processed, so the classification effect of the data level fusion approach is inferior to that of the decision level fusion approach.

### 3.3. Comparison of the fixed weight setting and the dynamic weight setting

**FIGURE 4:**
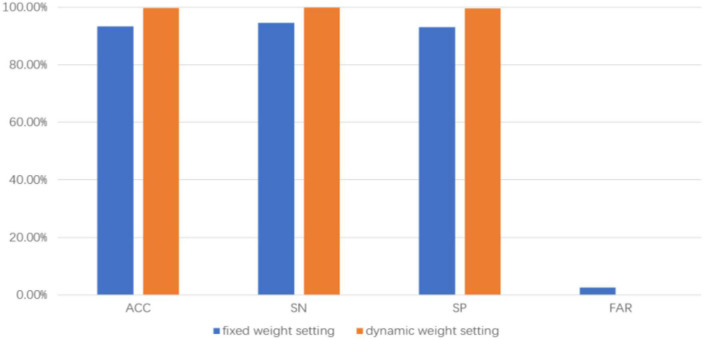
Comparison results with and without dynamic weight setting.

The proposed approach adopts the dynamic setting of the output weights of each branch network, which is based on the error rate of each branch. To verify the superiority of the dynamic weight method, the output weights of the two branches are fixed at 0.5, and the average accuracy, average sensitivity, average specificity, and average error alarm rate are 93.35%, 94.46%, 92.98%, and 0.026, respectively. When compared with the proposed approach, the approach of dynamically setting the output weight of each branch is better than the approach of fixedly setting the output weight of the branch network. The comparison result of the two approaches is shown in Figure 4.

### 3.4. Comparison of results of adversarial training and non-adversarial training

**FIGURE 5:**
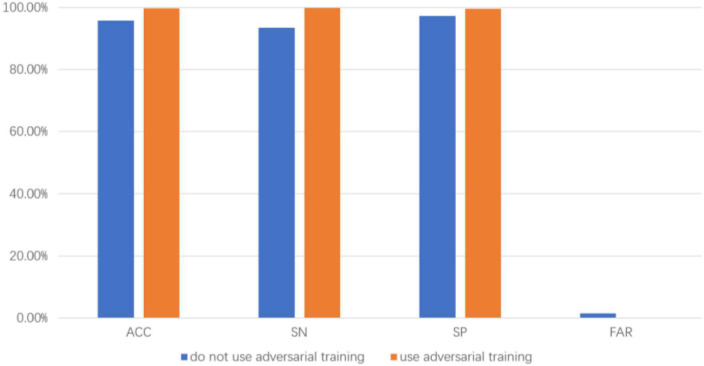
Comparison results with and without adversarial training.

In order to eliminate the negative impact of data difference of each episode in a patient, we adopt adversarial training to improve the generalization ability of the network, as a comparison method, the GRL and domain adversarial network are removed, the average accuracy, average sensitivity, average specificity and average FAR of eight epilepsy patients were 95.68%, 93.44%, 97.21% and 0.015, respectively, and by comparing with the proposed approach, the result shows that using adversarial training has better prediction performance. The comparison result of the two approaches is shown in Figure 5.

### 3.5. Advantages and limitations

This approach has the following advantages:

1. The proposed approach adopts the decision level data fusion strategy, the results of EEG and ECG data classification are combined, in the combining process, the classification error rate of each branch is used to calculate the weight, the branch with low error rate is set by a high weight, the branch with high error rate is set by a low weight, so that the network branch with low classification error rate plays a leading role in prediction, and the result of the network branch with high prediction error rate is used as a supplement, thus improving the prediction accuracy.
2. Since epilepsy data are time-varying, with differences between each seizure in a patient, this approach adopts domain adversarial training to extract the invariant features, thereby improving the generalization ability of the model.

This approach has limitations:

The proposed approach requires a dataset with both EEG and ECG signals, but there are few public datasets that meet this condition. The performance of the approach on multiple datasets cannot be demonstrated, so it is necessary to collect epilepsy data with both EEG and ECG signals from the local hospital for the demonstration.

## 4. Conclusion

In this work, a novel patient-specific approach to seizure prediction based on data fusion and adversarial training is proposed. To improve the prediction accuracy and robustness of the network, the data fusion strategy is adopted. To eliminate the instability of the network caused by the negative impact of data differences of each episode in a patient, domain adversarial training is adopted to extract the invariant features of a specific patient. We use the Siena scalp EEG dataset and leave-one-out cross-validation strategy, the average accuracy, average sensitivity, average specific and average FAR on the 8 epilepsy patients are calculated, our results outperform the competitive state-of-the-art baselines. In addition, the advantages of this approach are verified by comparing it with different data fusion methods and the use of domain adversarial training methods.

For future work, it is necessary to use techniques such as domain generalization to reduce the differences between patients for the clinical application of epileptic seizure prediction, and to develop a portable system that includes a wearable electrode cap and a smart device for epileptic seizure prediction.

## Data availability statement

Publicly available datasets were analyzed in this study. This data can be found here: https://physionet.org/content/siena-scalp-eeg/1.0.0.

## Ethics statement

Ethical review and approval was not required for the study on human participants in accordance with the local legislation and institutional requirements. Written informed consent from the participants’ legal guardian/next of kin was not required to participate in this study in accordance with the national legislation and the institutional requirements.

## Author contributions

YY and FL conceptualized the study. YY and XQ performed the methodology. YY and HW accounted the software. YY and XL wrote and prepared the original draft. XQ reviewed and edited the written draft. All authors read and agreed to the published version of the manuscript.
